# Effective Aging Inhibition of the Thermoplastic Corn Starch Films through the Use of Green Hybrid Filler

**DOI:** 10.3390/polym14132567

**Published:** 2022-06-24

**Authors:** Di Sheng Lai, Azlin Fazlina Osman, Sinar Arzuria Adnan, Ismail Ibrahim, Midhat Nabil Ahmad Salimi, Awad A. Alrashdi

**Affiliations:** 1 Faculty of Chemical Engineering Technology, Universiti Malaysia Perlis (UniMAP), Arau 02600, Perlis, Malaysia; disheng.lds@gmail.com (D.S.L.); sinar@unimap.edu.my (S.A.A.); ismailibrahim@unimap.edu.my (I.I.); nabil@unimap.edu.my (M.N.A.S.); 2 Biomedical and Nanotechnology Research Group, Center of Excellent Geopolymer and Green Technology (CEGeoTech), Universiti Malaysia Perlis (UniMAP), Arau 02600, Perlis, Malaysia; 3 Chemistry Department, Umm Al-Qura University, Al-Qunfudah University College, Al-Qunfudah Center for Scientific Research (QCSR), Al Qunfudah 21962, Saudi Arabia; aarashdi@uqu.edu.sa

**Keywords:** thermoplastic starch, nanocellulose, bentonite, biocomposite, aging, retrogradation

## Abstract

Recently, hybrid fillers have been widely used to improve the properties of biopolymers. The synergistic effects of the hybrid fillers can have a positive impact on biopolymers, including thermoplastic corn starch film (TPCS). In this communication, we highlight the effectiveness of hybrid fillers in inhibiting the aging process of TPCS. The TPCS, thermoplastic corn starch composite films (TPCS-C), and hybrid thermoplastic corn starch composite film (TPCS-HC) were stored for 3 months to study the effect of hybrid filler on the starch retrogradation. TPCS-C and TPCS-HC were prepared by casting method with 5 wt% of fillers: nanocellulose (NC) and bentonite (BT). The alteration of the mechanical properties, aging behavior, and crystalline structure of the films were analyzed through the tensile test, Fourier transform infrared (FTIR), X-ray diffraction (XRD), differential scanning calorimetry (DSC), and water absorption analysis. The obtained data were correlated to each other to analyze the retrogradation of the TPCS, which is the main factor that contributes to the aging process of the biopolymer. Results signify that incorporating the hybrid filler (NC + BT) in the TPCS/4BT1NC films has effectively prevented retrogradation of the starch molecules after being stored for 3 months. On the contrary, the virgin TPCS film showed the highest degree of retrogradation resulting in a significant decrement in the film’s flexibility. These findings proved the capability of the green hybrid filler in inhibiting the aging of the TPCS.

## 1. Introduction

The world is emphasizing the consumption of green energy and material for sustainability, thus biopolymers such as starch, polylactic acid, chitosan, and others are intensively developed to become the next potential material to replace the conventional plastics [1]. Starch-based biopolymers are highly attractive due to their high abundance on the earth, being environmentally friendly, and having the same process-structure property as fossil-fuel plastic, which makes them viable as packaging materials [2]. Starch is semi-crystalline and made up of two different macromolecules: amylose and amylopectin. The ratio of amylose to amylopectin varies according to the botanical origin of starch. The properties of the starch and starch-based plastic are strongly dependent on the ratio of these two macromolecules [3]. Typically, except for waxy starch and high amylose starch, starch will contain a higher amount of amylopectin (70–80%) than amylose (20–30%) [4]. Amylopectin is highly branched, while amylose possesses a linear molecular structure. The crystalline structure of starch is mainly contributed by double helix branched amylopectin. The neighboring branching of amylopectin chains with a degree of polymerization from 10 to 20 forms a double helix structure, causing the formation of high-order allomorphs. The interactions between double helix amylopectin and long chain amylose give rise to the semi-crystalline structure in the starch biopolymer. The alternate arrangement of the double helix crystalline lamella and the amylose amorphous lamella is known as a “growth ring”, which becomes the basic structure of the starch granules. 

It is clearly understood that the pristine form of starch has a poor processing ability due to an intense and strong hydrogen bonding network structure. Commonly, starch will degrade before reaching its melting temperature; therefore, plasticizers such as water, glycerol, polyol, and urea are required to weaken and break down the hydrogen bonding by forming the new hydrogen bonding with the plasticizer. Under shearing force, high temperature, and incorporation of plasticizers, granule starch can be processed into an amorphous and homogenous material known as thermoplastic starch (TPS). Glycerol and water are the most common plasticizers as they are compatible with the starch structure and were proven in many studies [5,6]. Glycerol and water can effectively reduce the intermolecular force of granule starch and loosen up the dense packing structure. Full disruption of the starch structure can be achieved through the presence of plasticizers and a sufficient supply of thermomechanical energy [7]. 

Virgin TPS films are rarely utilized due to their low mechanical properties and high moisture sensitivity. The high moisture absorption of the virgin TPS is due to the existence of a large number of hydroxyl groups in the starch structure [8]. Besides that, the metastability of the TPS structure may lead to recrystallization or retrogradation process during storage over a period. Retrogradation can deteriorate the characteristics of the TPS films as it will alter the mechanical properties and affect the quality of the final product. Consequently, this will limit the usage of TPS in many applications.

Many factors can lead to the retrogradation of the TPS films, such as humidity, storage time, and glass-transition temperature, Tg [7]. Retrogradation is a process of reconstruction of the crystalline structure of starch chains. Despite the controversy about the accurate mechanisms of retrogradation that happen in TPS films, retrogradation was identified as the main cause of the aging and brittleness of this bioplastic. Generally, the retrogradation process can be divided into three stages, (i) nucleation, (ii) propagation (growth of crystalline structure), and (iii) maturation (perfection of crystals) [8]. Two retrogradation processes occur during the aging of the TPS films: linear amylose structure recrystallizes into a single helix structure (fast retrogradation rate) and branched amylopectin recrystallizes into a double helix structure (slow retrogradation rate). The slow retrogradation of amylopectin compared to amylose is due to its limited chain dimensional and lower crystalline structure stability [8]. The retrogradation rate was highly dependent on the mobility freedom of starch molecules. The restoration of crystallinity structure, loss of water holding capacity, and increased stiffness of the films are the most commonly used methods to determine the retrogradation of the TPS films [9]. A large amount of research was conducted to prevent starch’s retrogradation by incorporating different types of plasticizers such as glycerol, polyol, urea citric acid, formamide, and so on to reduce the interaction between the starch chains [9,10]. A plasticizer can be defined as a substance (typically a solvent) added to synthetic or natural polymers (biopolymers) to reduce brittleness by promoting plasticity and flexibility. Several papers have discussed the role of mono-plasticizer and co-plasticizer in controlling the retrogradation of the TPS films. The co-plasticization method is preferred to produce the TPS films as it can compensate for the disadvantages of each plasticizer to make better properties films compared to mono-plasticizer films. For instance, Esmaeili et al. employed glycerol and sorbitol as co-plasticizers to form the TPS films. The low mechanical strength of glycerol/TPS films was successfully enhanced by incorporating sorbitol. However, the film’s ductility was greatly reduced by 80% compared to the glycerol/TPS films [11]. In another work, Khan et al. have proposed the use of boric acid and glycerol as co-plasticizer to reduce the starch retrogradation. They showed that the boric acid can provide a better anti-retrogradation effect compared to the glycerol, whereas the TPS films that contain co-plasticizers have higher moisture sensitivity compared to glycerol/TPS films [12]. Glycerol was also proposed to be a co-plasticizer with other components containing amide groups such as urea, formaldehyde, and formamide in order to reduce the retrogradation in the TPS by forming a stronger hydrogen bonding [13,14,15]. Nonetheless, this kind of plasticizer is rather toxic to human health; therefore it is not suitable to be applied in food packaging material. Several researchers have proposed the incorporation of a co-plasticizer that can form crosslinking with the TPS chains such as citric acid, malic acid, and choline salts–DES to reduce the retrogradation of the TPS [16,17]. However, the ductility of the co-plasticizer films was significantly reduced, making them brittle and unsuitable for film packaging applications. Therefore, researchers have investigated the incorporation of additives into the TPS structure, such as filler or nanofiller to restrict the mobility of the TPS chains. Nanofillers are those fillers that have one of their dimensions in the nano-size range (less than 100 nm). Nanocelluse and nanoclays such as bentonite and montmorillonite are examples of the nanofiller that can be used to reinforce and improve the properties of the TPS films. The solid and compact interfacial bonding between the filler/nanofiller and the TPS chains may form a highly stable thermodynamic 3D networking structure that can prevent retrogradation [18]. 

The crystalline structure of starch acts like a cement structure embedded in the amorphous regions, increasing the films’ stiffness, and reducing the elongation at the break of the films. Starch’s retrogradation happens during storage by forming hydrogen bonding with its adjacent starch chains. Therefore, incorporating nanocellulose and bentonite is expected to interfere with the alignment of the starch chains, thus preventing them from “self-interactions”. Lendvoi et al. have successfully reduced the retrogradation of TPS starch through the incorporation of bentonite. Intercalation of bentonite in between the TPS molecular chains has successfully suppressed the retrogradation of starch, however, at the cost of the flexibility of the films [19]. Balakrishnan et al. studied the effect of nanocellulose on the properties of the TPS films. They have concluded that the nanocellulose confined the chain segment of the TPS chains, reducing the mobility of the starch macromolecules and suppressing the retrogradation of starch during aging [20]. Several review papers have included more detailed information on the effect of nanofillers on the aging of the TPS films [6,21,22]. Moreover, the size and number of the hydroxyl group of the filler were proposed to play a significant part in preventing the retrogradation of starch. Meanwhile, some researchers found out that the hydroxyl group’s reactivity and bond-forming ability are more important in forming a stable hydrogen bond with a starch chain to prevent retrogradation. Nevertheless, most of the research concluded that the prevention of retrogradation depends on the hydrogen strength forming between the starch, fillers, and plasticizers. The stronger the hydrogen formed between the starch chain, fillers, and plasticizer, the slower the retrogradation rate [22]. 

Recently, hybrid fillers have been studied as potential alternative components to inhibit the retrogradation in TPS films. TPS films produced by hybrid fillers may combine the good properties of both fillers and optimize the best mechanical performance for the films. The hydrophilic type of fillers, such as nanocellulose and nano-bentonite, are compatible with the TPS, which is also hydrophilic. Hybrid fillers were studied extensively to enhance the mechanical properties of TPS films. It has the vast potential to develop into packaging material. Many research papers studied the effect of hybrid filler on the TPS films but mainly focused on the mechanical strength, structure, and humidity properties [1,23,24]. There is still a lack of studies and reports on the relationship between hybrid fillers on the retrogradation rate of TPS films due to time-consuming data collection. The effect of storage time on the properties of the TPS hybrid biocomposite is somewhat limited. Our previous study found that a hybrid of nanocellulose (NC) and nano-bentonite (BT) has a synergy effect in enhancing the toughness of the TPS films [25]. Thus, to close the research gap in this field, this current work aims to analyze the effect of green hybrid filler addition (NC + BT) on the TPS film’s retrogradation during aging. In this study, corn starch powder was utilized as raw material. Corn starch is a type of starch that is derived from maize (corn) grain, which can be obtained from the kernel and endosperm part of the plant. In this article, thermoplastic starch derived from corn is referred to as thermoplastic corn starch (TPCS). The impact of hybrid filler on the TPCS film’s mechanical property was studied throughout the storing period. A tensile test was applied to observe the mechanical stability throughout the 3-month storage. DSC, XRD, and FTIR analyses were employed to study the crystalline structural change, enthalpy crystalline melting, and crystalline melting temperature of the TPS films. These three tests are highly sensitive and have been proven in many studies as valuable and reliable tools to quantify the retrogradation that happens in TPS films [15,16]. All the data were compiled, analyzed, and correlated with the retrogradation process of the starch chains, to investigate the effectiveness of the hybrid filler in inhibiting the aging of the TPCS matrix.

## 2. Experimental

### 2.1. Materials

Corn starch (72% amylopectin, 28% amylose) was purchased from Sigma Aldrich (St. Louise, MO, USA). It was employed as a matrix material after being plasticized into thermoplastic form. The plasticized corn starch is referred to as thermoplastic corn starch (TPCS). The nanocellulose and bentonite were combined to form a hybrid filler (NC + BT) of the TPCS biocomposite film. The nanocellulose was extracted from the oil palm empty fruit bunch (OPEFB) fiber purchased from United Oil Palm Industries Sdn Bhd (Nibong Tebal, Malaysia). Natural nano-bentonite clay was obtained from Sigma-Aldrich (St. Louise, MO, USA). Details on the hybrid filler preparation can be found in our previous paper [14]. Glycerol was purchased from HmbG Chemicals (Hamburg, Germany). It was used as a plasticizer with deionized water.

### 2.2. Preparation of TPCS, TPCS-C, and TPCS-HC Films

The virgin TPCS films, TPCS biocomposite films (TPCS-C), and hybrid TPS biocomposite films (TPCS-HC) were prepared through the casting method. A total of 5 g of corn starch was dispersed in 100 mL distilled water and a 2 g glycerol mixture to form thermoplastic corn starch films. The mixture was kept stirring at 300 rpm using a heated magnetic stirrer for 30 min at 80 °C to obtain homogenous TPS gel. For TPCS-C and TPCS-HC, 5 wt% of single filler or hybrid filler were prepared and then subjected to an ultrasonication process before being incorporated into the TPCS matrix. There were two ratios of hybrid fillers selected: (i) 4BT:1NC and (ii) 2BT:3NC for producing the TPCS biocomposite films. These two ratios were selected based on our previous study where 4BT:1NC was found to be the optimum ratio to produce the toughest films, while 2BT:3NC was observed to result in the lowest toughness value to the TPCS-HC film.

In the preparation of the biocomposite films, the mixture of TPCS/BT/NC was continuously stirred for 10 min to achieve homogenous dispersion of hybrid filler in the matrix. The homogenous TPCS suspension was poured into an 8-inch round Teflon casting plate and dried in the oven for 24 h at 45 °C. The composition and the abbreviation of the samples are presented in Table 1.

#### Storage Procedures for Aging Analysis

**Figure 1:**
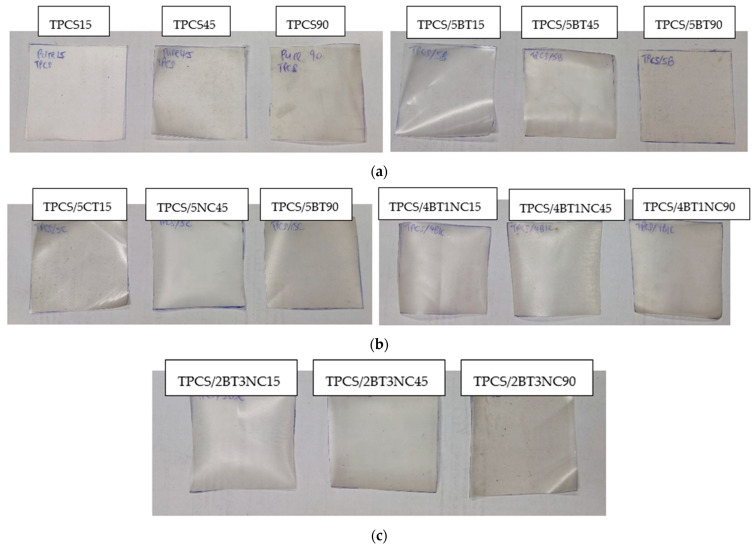
The physical appearance of virgin TPCS, TPCS-C, and TPCS-HC films aged for (**a**) 15, (**b**) 45, and (**c**) 90 days.

The TPCS, TPCS-C, and TPCS-HC films were kept in a chamber with a constant humidity of 53% at 25 °C and stored for 15, 30, 45, 60, and 90 days. The film samples were taken out for testing and analysis after the specified storage period. Figure 1 shows the films’ appearance after being stored for 15, 45, and 90 days.

### 2.3. Testing and Characterization of Films

#### 2.3.1. Tensile Test

A tensile test was carried out according to ASTM-638 Type V to determine the tensile properties of films with different storage duration using the Universal Instron Machine model-5582 (Norwood, MA, USA). The tensile test was performed with a 5 kN load sensor and 10 mm/min crosshead speed. The testing was carried out at room temperature (23 °C) with 53% humidity. Seven replicates were tested and the average values of tensile properties (tensile strength, Young’s modulus, and elongation at break) were acquired from the Instron Merlin software Version 5.41.00 (Instron^®^, Norwood, MA, USA) and then recorded.

#### 2.3.2. Differential Scanning Calorimetry (DSC)

Differential scanning calorimetry (DSC) analyses were carried out by TA instrument Q-10 (Lukens Drive, New Castle, DE, USA) to determine the rate of retrogradation of the TPCS-C and TPCS-HC. A total of 30 mg of samples were tested and analyzed between 25 °C to 180 °C with a heating rate of 10 °C/min in a nitrogen atmosphere to determine the enthalpy of crystallization of each sample. The reference pans used were 40 μL aluminum pans. According to ASTM D3418-03, the enthalpy of the crystalline structure was determined by measuring the area of the main endothermic peak, while the melt temperature was determined as the midpoint of onset and end temperature of the endothermic peak. The enthalpy change in temperature was calculated and identified by using TA universal analysis 2000 software version 4.5A (TA-instruments-Water L.C.C, Lukens Drive, New Castle, DE, USA). All the films were stored under the same humidity (53%) for 15, 45, and 90 days.

#### 2.3.3. X-ray Diffraction (XRD)

The crystalline structure of all the films was determined using the Bruker D2 Phaser X-ray diffractometer (Billerica, MA, USA) using Cu Kα X-rays. The samples were tested by using a scan rate of 0.1 s per step from 2θ = 10 − 40°. The analysis and identification of the peak was analyzed by using X’Pert HighScore plus under PANalytical B.V. Almelo, The Netherlands. Meanwhile, the calculation for the degree of crystallinity was performed using Originpro 2019 (b) under OriginLab Corporation Northampton, Massachusetts, USA. The testing was carried out at room temperature, 25 °C with 53% humidity. The crystallinity of the films was determined by calculating the subtracting the amorphous halo from the XRD scan shown in Formulation (1)

(1) $$\mathrm{Degree}\;\mathrm{of}\;\mathrm{crystallinity}\;(\%)=\frac{I_{o}-I_{a}}{I_{o}}\;\times \;100\%$$

where I_o_ = Total surface area of the peak from the XRD-based line, I_a_ = Amorphous halo from the XRD scan.

#### 2.3.4. Fourier Transform Infrared (FTIR)

TPCS, TPCS-C, and TPCS-HC films were scanned by using a Perkin Elmer spectrum 65 (Waltham, MA, USA) FTIR spectrometer with a wavelength range of 650–4500 cm^−1^, 16 scans and resolution of 4 cm^−1^. All the films were stored at the humidity of 53%, 25 °C, and stored for 15, 45, and 90 days before conducting the FTIR analysis. The infrared spectra range of 900–1200 cm^−1^ was focused to calculate the intensity ratio of the spectra at a specific wavenumber (1045 cm^−1^:1022 cm^−1^). The purpose was to study the short recrystallize structure of the films. The infrared spectrum range 900–1200 cm^−1^ was deconvoluted, whereas the intensity ratio for 1045 cm^−1^:1022 cm^−1^ was calculated by using the Origin 2019 (B) software (OriginLab Corporation Northampton, MA, USA).

#### 2.3.5. Moisture Absorption

The moisture absorption test was determined by using the samples of 10 × 10 × 0.2 mm dimension to quantify the amount of moisture absorption during the 3-month storage duration. All the produced films were kept in the sealed humidity chamber with a control relative humidity (RH) of 53% at 25 °C. The film samples were immediately weighed after being taken out from the humidity chamber, and weighed in a specific period. The moisture content of the films was calculated by using Equation (2):

(2) $$\mathrm{Moisture}\;\mathrm{content}\;(\%)=\frac{W-W_{o}}{W_{o}}\;\times \;100\%$$

where W = the film’s weights after taking out from the humidity chamber and W_o_ are the original mass of the films right after the films form. The test was repeated 3 times to obtain the average value of the moisture content [15].

## 3. Results and Discussion

### 3.1. Mechanical Analysis of TPCS, TPCS-C, and TPCS-HC Films Aged for 15, 30, 45, 60, and 90 Days

#### 3.1.1. Tensile Strength

**Figure 2:**
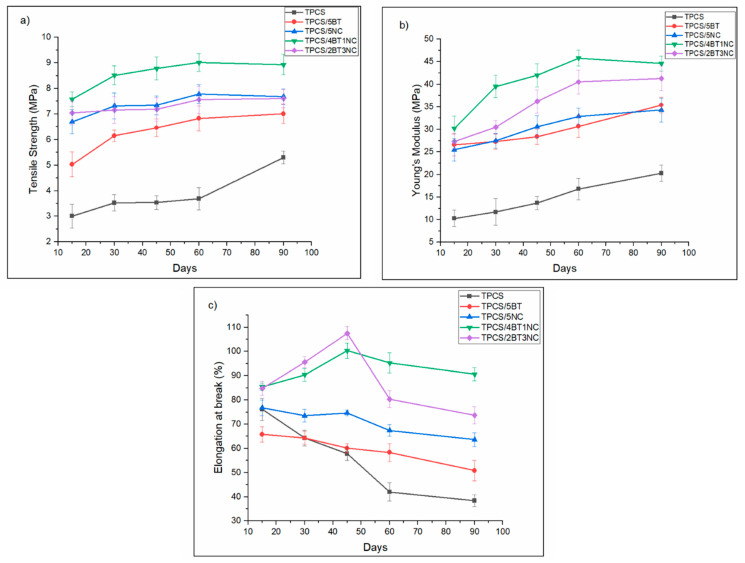
(**a**) Tensile strength, (**b**) Young’s modulus, and (**c**) Elongation at break of TPS, TPSC, and HTPS films aged for 15, 30, 45, 60, and 90 days.

Measuring the change of mechanical properties across a period is one of the effective ways to postulate the aging properties. Figure 2 summarizes the mechanical properties of the TPCS, TPCS-C, and TPCS-HC biocomposite films for 15, 30, 45, 60, and 90 days of storage. After being stored for 3 months, the tensile strength of the virgin TPCS increased from 3.01 MPa to 5.3 MPa, almost 76% during the storage. The gradual increase of the tensile strength indicates the retrogradation of the TPCS occurred due to enhancement in crystallinity, as detected through XRD and DSC analyses. More crystalline structure in the starch chains requires greater force to break the molecular bonds; therefore the tensile strength value increases [26]. Meanwhile, the tensile strength of the TPCS/4BT1NC sample has been slightly increased (from 7.05 MPa (15 days) to 8.31 MPa (90 days)) throughout the storage period.

Generally, the trend shown in these tensile test data can be attributed to the structural evolution of the TPS, which is also influenced by the plasticizer and hybrid filler. Tensile strength for all the films increases in the first 15 days to 30 days, indicating migration of plasticizers and rearrangement of starch microstructure that occur within the first 30 days. This observation was in accordance with previous studies, and those results were proved by the XRD analysis which indicated the increase in the crystalline structure of the host TPS in the first month of storage. An increase in the crystallinity was attributed to the migration of plasticizers, resulting in an increase in tensile strength [27,28,29]. However, the tensile strength of the TPCS/4BT1NC was relatively stable after the first 30 days compared to other films. This shows that the interaction between TPCS, plasticizer, and hybrid filler could effectively minimize the rearrangement of the starch molecular chain. TPCS/5BT and TPCS/5NC showed a gradual and constant increase in strength with increased storage time. Meanwhile, the TPCS films show a noticeable increase in tensile strength after 60 days. The rise of the TPCS films’ tensile strength was contributed by the increased crystallization of the amylopectin structure in the TPCS matrix, as shown in DSC and XRD analyses. The increase of the recrystallized amylopectin region indicated that the 3D starch network formed was less stable compared to other films under the normal storage condition. The absence of filler/hybrid filler caused the migration of the plasticizer to the film’s surface. The void left behind by the plasticizer causes amylopectin to form bonding with the adjacent molecular chains and form another crystalline structure.

It is worth mentioning that the retrogradation rate of the TPCS films is highly dependent on the TPCS’s chain mobility. Strong interfacial bonding within the TPCS chains can reduce the starch chain mobility and thus, effectively hinder the retrogradation. The stronger the interfacial bonding, the slower the retrogradation rate. Therefore, incorporating a hybrid filler is postulated to reduce the mobility and retrogradation process of the starch chains, but at the same time increase the glass-transition temperature, Tg, of the TPCS. Meanwhile, for the virgin TPCS films, the high moisture absorption capability caused the decrease of Tg across the storage time. As the Tg decreases, the degree of plasticizer migration increases with the storage time [30]. Therefore, we can observe a sudden increase in tensile strength from 3.69 MPa (60 days) to 5.30 MPa (90 days). By comparing the tensile strength of the TPCS, TPCS-C, and TPCS-HC films for 3 months, the TPCS-HC was seen to show the best retention in the tensile strength value, suggesting that the hybrid fillers can effectively slow down or limit the retrogradation through solid interaction between the plasticizer and TPCS chains.

#### 3.1.2. Young’s Modulus

The Young’s modulus trend is almost identical to tensile strength, where the TPCS films show the highest increase in Young’s modulus after 90 days of storage, even though the value is still the lowest among all the samples. The virgin TPCS film exhibits a 100% increase in Young’s modulus from 10.1 MPa to 20.3 MPa after 3 months of storage. Meanwhile, the TPCS/4BT1NC demonstrates the lowest increase of Young’s modulus (20% increase of Young’s modulus from 37.2 MPa to 44.6 MPa). By comparing the increment percentage, it can be postulated that the TPCS/4BT1NC has relatively higher structural stability than other films. The progressive increase of the TPCS film’s Young’s modulus can be attributed to the retrogradation. During the storage, the amylose and amylopectin recrystallize into a single helix and double helix crystal structure through the expulsion of plasticizers located within their adjacent chains. This enhances the crystallinity of the TPCS and leads to the stiffening and brittleness of the TPCS film [31,32]. Obviously, the modulus of the TPCS/4BT1NC was rather stable throughout the storing period, indicating there was no drastic alteration of the crystalline structure causing the fluctuation in Young’s modulus value. 

The Young’s modulus of TPCS/2BT3NC has shown a different trend than its tensile strength. The tensile strength of the TPCS/2BT3NC showed a slight decrease from days 45 to 90. However, the Young’s modulus value showed a slightly increased from 32.2 MPa to 38.3 MPa. The slightly increased Young’s modulus indicating the less chain mobility of TPCS/2BT3NC can be associated with the increased crystalline structure as proved in XRD analysis. However, DSC showed that the crystalline structure in TPCS/2BT3NC is highly unstable, and low energy is required to break the crystalline structure. Therefore, the increase in crystalline structure is not reflected in the tensile strength. 

TPCS/5BT and TPCS/5NC biocomposites demonstrate a gradual increase of Young’s modulus through the storage time. The increase of the modulus can be due to the increase in the crystalline structure of the TPCS-C chain or can be related to the improved reinforcing effect of filler with increased storage time. As the migration of plasticizers happens during storage, it will trigger the recrystallization in TPCS-C and increase the film’s stiffness. The migration of plasticizers will promote a “closer” interaction between the filler and starch chains, which can improve direct interfacial interaction and form the glassy structure in the TPCS matrix [15].

#### 3.1.3. Elongation at Break

The elongation at break of the TPCS films has significantly decreased from 76.1% to 38.4% and became highly brittle as the storage time increased. The embrittlement of the TPCS films was one of the undesired characteristics caused by the retrogradation. It has been well documented that the amorphous structure of amylose and amylopectin can recrystallize into the different crystalline structures (single-crystalline or double helix crystalline structure) during the storage [3,16]. The different crystalline structure formed in TPCS films will cause heterogeneous phases occurs as amylose and amylopectin recrystallize through intra- and intermolecular bonding. The different extent of chain recrystallization rate and crystalline structure induces the hard and soft segment order in the TPCS matrix. The disparity hardness between these segment orders could increase the internal stress of TPCS films at the junction of crystalline structure when the film is stretched. The internal stress evolves into the microvoids, which results in premature failure and reduction in the elongation at break [16]. 

The elongation at the break of the TPCS/4BT1NC was gradually increased from 85.5% (15 days) to 112.3 % (30 days) but then decreased slightly to 92.6% (90 days). Interestingly, the TPCS/4BT1NC does not show a significant reduction in the elongation at break value compared to other films, indicating the hybrid fillers could stimulate stress relaxation in the hard segment order and enhance the soft segment order which reduces the internal stress build-up in the films as mentioned in our previous study [24]. Thus, the TPCS/4BT1NC is physically stable across the storage and aging. 

Apparently, the results suggest that the stability toward the aging of the TPCS/2BT3NC film was less prominent as compared to the TPCS/4BT1NC film. The TPCS/2BT3NC exhibits a stable microstructure for the first 60 days; however, as the storage increased to 90 days, the microstructure stability was reduced, and retrogradation was detected after reaching 60 days. This shows that the ratio of NC/BT filler in the hybrid system would affect the long-term retrogradation of the TPCS films. It can be said that the high composition of the NC compared to BT in the biocomposite films can attract the migration of plasticizers toward the NC. The long storage period has caused an accumulation of plasticizers in the interface of NC and the amylopectin. This encouraged the crystallization of the amylopectin chains, forming the unstable transcrystalline region in the starch structure [33,34]. At the same time, the number of the plasticizer molecules in the amylopectin-rich region decreased, leading to reduced chain mobility and decreased elongation at break. As a result, elongation at break for the TPCS/5BT and TPCS/5NC films decreased along with the increase in storage time. However, the elongation at break for both films is still better than the virgin TPCS films, indicating that the single filler (BT or NC) can prevent retrogradation of the TPCS matrix to some extent, although not as good as the hybrid NC/BT filler.

### 3.2. Thermal Analysis for TPCS, TPCS-C, and TPCS-HC Films Aged for 15, 45, and 90 Days

**Figure 3:**
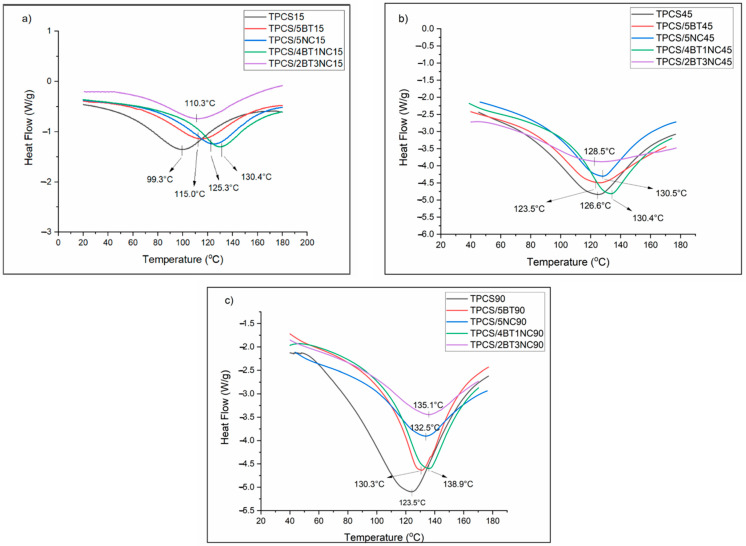
DSC heating curves of virgin TPCS, TPCS-C, and TPCS-HC films aged for (**a**) 15, (**b**) 45, and (**c**) 90 days.

Figure 3 presents the DSC thermal analysis of the virgin TPCS, TPCS-C, and TPCS-HC biocomposite films, which were stored for 15, 45, and 90 days. This thermal analysis was performed to study the thermal transition throughout the 3-month storage time. The thermal transition was detected by measuring the heat flow differences between the TPCS films and the reference pans, which can usually be related to the melting of the crystalline structure in the films.

The thermal transitions of the virgin TPCS and associate biocomposites were analyzed by observing the changes in the enthalpy of fusion of the melting endotherms (ΔHf) and melting temperature (T_m_). The recrystallized structure of the retrograded starch films can be realized through the observed enthalpy changes. The increase of enthalpy melting can be related to the extent of recrystallization happening in the TPCS structure. The homogeneity and the quality of the crystallized structure formed by starch retrogradation also can be studied by the broadness and the intensity of the melting peak. A more complex, broad, and weak melting peak can be associated with a high number of the different crystalline structures formed in the matrix with varying morphologies. By comparing the DSC heating curves between the TPCS, TPCS-C, and TPCS-HC films, we can reveal the effect of single filler and hybrid filler on the recrystallization structure of TPCS. The melting temperatures (T_m_) and enthalpy of fusion (ΔH_f_) for TPCS, TPCS-C, and TPCS-HC films are summarized in Table 2, while the DSC heating curves are shown in Figure 3. 

During the first 15 days, all the samples exhibited a broad and weak endothermic melting peak. TPCS15 films showed the highest enthalpy of melting (194.3 J/g) followed by TPCS/2BT3CN15 (186.6 J/g), TPCS/4BT1NC15 (178.5 J/g), TPCS/5BT15 (142.6 J/g) and TPCS/5NC15 (135.4 J/g). Even though the TPCS films have the highest melting enthalpy, it possesses the lowest T_m_ (99.3 °C) and a broad endothermic peak, suggesting that the crystalline structure in the starch is non-uniform and less stable compared to other films. During the retrogradation process, the TPCS chains may form different structures and morphologies and result in distinct retrograded crystallites [35]. The differences of distinct retrograded crystalline structures may cause the different extent of the crystalline structure formed in the TPCS matrix and result in the large and broad endothermic peak being detected. Meanwhile, incorporating single or hybrid fillers into the TPCS has increased the T_m_ of the crystalline structure. Even though incorporating the BT and NC can hinder the recrystallization in the TPS films, studies also showed that the BT and NC may provide the “thermal shielding effect” to the matrix phase, which causes a higher melting temperature needed to break the structure [24,36]. Moreover, the difference in morphology between the BT and NC may contribute to different ways of matrix-filler interactions, which causes a difference in T_m_ between the TPCS/5BT15 and TPCS/5NC15.

As the storage time increased to 45 days, the T_m_ of the TPCS, TPCS-C, and TPCS-HC films increased, and the endothermic peak became sharper and more intense. The T_m_ of TPCS/4BT1NC45 only shows a small increase (+4 °C), but the melting enthalpy showed a tremendous increase from 178.5 to 412.4 J/g. The significant growth of enthalpy could be due to the enhancement effect of hybrid fillers toward the crystalline structure which required higher thermal energy to melt the crystalline structure [24]. The melting temperature and enthalpy of the TPCS films also significantly increased by as much as 24.3 °C and 241.3 J/g from day 15 to 45. The increase of both parameters indicated the crystalline structure continued to develop into a more well-defined and stable crystal structure. Interestingly, the T_m_ of the TPCS/2BT3NC45 was only increased by 18.2 °C. However, it shows only a small increase of melting enthalpy (128.5 J/g) from day 15 to 45 days. The small increment and broad melting enthalpy peak of TPCS/2BT3NC45 indicate that the crystalline structure formed in TPCS/2BT3NC45 is highly unstable and non-uniform in size. 

As the storage time increased to 90 days, the T_m_ and melting enthalpy of the films increased and become more well defined. The melting peak of the TPCS90 becomes broader and more intense compared to other films. The high-intensity TPCS90 melting peak can be related to a high retrogradation rate due to the high growth rate of the crystal structure. However, the broader peak observed could be due to the high number of imperfect and non-uniform crystals [23,37]. The higher Tm and sharper melting enthalpy of the TPCS/5NC90 and TPCS/5BT90 compared to the TPCS90 indicate that a more uniform and stable crystalline structure can be induced by adding NC or BT. Even though NC and BT can induce crystalline structure forming in the TPCS matrix, the overall increase in the melting enthalpy of TPCS/5NC and TPCS/5BT is rather smaller than the TPCS films from days 45 to 90. This shows that BT and NC can reduce the retrogradation of starch for a prolonged storage period. The DSC heating curve of the TPCS/2BT3NC90 revealed the lowest melting enthalpy of this sample compared to other samples, after 3 months of storage. The low melting enthalpy indicates that there is less thermal energy required to destroy the crystalline structure of the TPCS/2BT3NC90. Furthermore, the fluctuation in the melting temperature of the film suggests that the retrogradation process occurred to some extent. Meanwhile, the T_m_ and melting enthalpy of the TPCS/4BT1NC show relatively stable across the 3-month storage period. The sharp and low intensity of melting enthalpy from the beginning of storage indicates the hybrid filler with a 4:1 ratio is highly favored to form a stable crystalline structure at the early stage of film-forming and restrict the mobility of the amylopectin, preventing the retrogradation phenomenon. 

### 3.3. Crystalline Structure of TPCS, TPCS-C, and TPCS-HC Films Aged for 15, 30, 45, 60, and 90 Days by Using XRD Analysis

**Figure 4:**
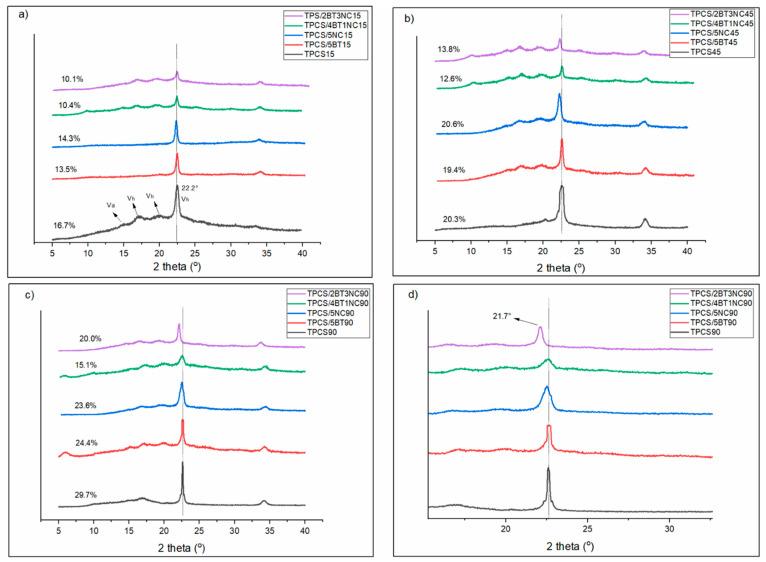
XRD scan for virgin TPCS, TPCS-C, and TPCS-HC films aged for (**a**) 15, (**b**) 45, and (**c**) 90 days from 2θ= 5.0–40.0° and (**d**) 90 days from 2θ = 10.0–30.2°.

Overall, the TPCS, TPCS-C, and TPCS-HC films’ crystallinity increased with the storage time. The longer the storage time, the higher the crystalline percentage. However, incorporating a single or hybrid filler slowed down or inhibited the retrogradation rate. Table 3 and Figure 4 shows the changes in the crystalline percentage of the TPCS, TPCS-C, and TPCS-HC films after 15, 45, and 90 days of aging. The virgin TPCS film demonstrates the highest rate of the crystals growth as the crystalline percentage reaches 28.7% in the 3-month storage. Meanwhile, TPCS/5BT and TPCS/5NC exhibit a lower crystallization percentage compared to the TPCS films. For TPCS-HC films, TPCS/2BT3NC and TPCS/4BT1NC proved to effectively prevent the retrogradation in starch for the first 45 days as the crystalline percentage only showed a minor increase of 3.7% and 2.2%, respectively. The crystalline percentage of TPCS/4BT1NC experienced a slight increase in crystallinity structure (2.9%) even though the storage period increased to 3 months. However, for TPCS/2BT3NC, the efficacy to inhibit retrogradation was only seen to be dominant in the first 45 days. This is because the crystallinity of the TPCS/2BT3NC increased drastically to 20% after 3 months of storage, showing that the retrogradation process has taken place.

The XRD diffraction pattern of the TPCS films, TPCS-C films, and TPCS-HC films that were stored for 15, 45, and 90 days was recorded. Upon the film drying process, the amylose chain will undergo fast retrogradation to form a Va-type or Vh-type crystalline structure with the inclusion of plasticizers, which is typically detected in 2θ = 7.8°, 15.0°, 20.8°, and 2θ = 17.0°, 18.3°, 22.1° respectively. However, the Va-type crystals are relatively unstable compared to the Vh-type crystalline structure; they would absorb water molecules and transform to the Vh-type crystalline during storage in ambient humidity [38]. Therefore, a small amount of Va-type crystalline residual structure was detected in TPCS films’ XRD diffraction pattern. The complete transformation of crystalline usually happens in the first 20 days [11,39,40]. The crystalline structure change by absorbing water molecules agreed with the water absorption result of the pure TPS films. As the storage time increased to 45 days, the residual for Va-type crystalline disappeared and completely transformed into Vh-type crystalline. Meanwhile, as the TPS films were scanned on day 45, the intensity of the Vh seemed to reduce. This phenomenon was explained by Schmitt et al. where the Vh-type crystalline structure was superimposition with B-type crystalline during the slow retrogradation process, causing the intensity of the Vh to be reduced in the XRD diffraction diagram [16].

Based on the XRD curves, the TPCS15 and TPCS/5NC15 and TPCS/5BT15 exhibited a firm Vh-type retrogradation peak compared to TPCS/4BT1NC15 and TPCS/2TB3NC15 for the first 15 days. The high intensity of the Vh structure strongly suggests the plasticizer was not effectively dispersed in the TPCS matrix, resulting in the formation of the Vh-plasticizer crystalline structure. Meanwhile, only a small Vh peak was detected in both TPCS-HC films, indicating that the hybrid filler more effectively interacts with the plasticizer and restricts the mobility of plasticizer molecules to prevent the formation of Vh-plasticizer crystalline structure. 

In the case of TPCS/5NC and TPCS/5BT, the increased intensity of the peak at 2θ = 22.2° over time can be due to the nucleating effect of the fillers. Generally, slow retrogradation consists of three major steps (nucleation, propagation, and maturation) in recrystallization [8]. The nucleation of the crystalline structure started at the early stage of film-forming. It was propagated throughout the storing time. The crystalline structure was finally matured after three months of storage and increased the intensity of the peaks. The biocomposite incorporating the single filler, regardless of BT or NT, exhibited a lower crystalline percentage than the TPCS films after 3 months, indicating that a single filler can suppress the retrogradation in starch. However, Lendvai et al. showed a contrary result where the addition of filler increases the retrogradation rate in starch. The increase in crystalline percentage may be attributed to poor filler dispersion in the TPS matrix. The agglomerated filler served as the nucleating site to facilitate the recrystallization of the starch chain [30]. 

The crystalline structure of the TPCS/4BT1NC only experienced slight changes after 3 months of storage, evidently proved by the slight alteration of the diffraction peaks. This result was also in agreement with the stable mechanical performance of the films. All the films exhibited a more defined peak as the storage time increased. A pronounced increase in peak intensity at 2θ = 22.2° was observed in TPCS, TPCS/5NC, and TPCS/5BT after 45 days of storage. However, the increased intensity of this peak was not observed in the TPCS-HC films, suggesting that the hybrid fillers can effectively form stable TPCS structures and reduce the retrogradation. However, as the NC ratio in the hybrid filler increased to 3, the peak intensity at 2θ = 22.2° for TPS/2BT3NC has a noticeable increase after 3 months of storage. This increase in peak intensity indicates that the semi-crystalline structure of the TPCS/2BT3NC is not stable with a long storage time. The inhibition of the retrogradation process is only prominent during the early storage period. TPCS/2BT3NC is only effective in preventing the fast retrogradation happening in starch, and it was less effective in suppressing the slow retrogradation happening in the TPCS films compared to the TPCS/4BT1NC. 

The effectiveness in inhibiting the retrogradation in TPCS/4BT1NC and TPCS/2BT3NC may be attributed to the different ratios of the hybrid filler component. The increase in NC ratio in the TPCS-HC films resulted in the low TPS film’s stability in long-term storage. Previous studies showed that the NC has a higher plasticizer affinity than the starch molecules. Drakopoulos et al. have studied the interfacial effect of TPS, microcrystalline cellulose, and plasticizer by using broadband dielectric spectroscopy (BDS). They showed that even though the microcrystalline cellulose and starch chains have high similarities in chemical structure, they possess different dimensional structures that affect their polarization toward plasticizers. The difference in dimensional structure makes the microcrystalline cellulose more hydrophilic, thus water molecules can more easily enter and decrease the polymer chain’s mobility [41]. As the storage time increases, the plasticizer in the TPCS/2BT3NC will slowly get away from the amylopectin-rich phases. The voids left behind by the plasticizer causes more water to be diffused into the amylopectin structure and facilitates the recrystallization. This result agrees with the water absorption data of the TPCS/2BT3NC as the water absorption of TPCS/2BT3NC is slightly decreased compared to the TPCS/4BT1NC after 3 months of storage. This explained the sudden increase in crystalline percentage and intensity peak of the TPCS/2BT3NC at 2θ = 22.2° after 45 days of storage. The unexpected crystalline alteration was also seen in the study of A. Ujcic et al. However, they showed the opposite trend where the crystallinity of the TPS composite films decreased in the middle of the storage period by incorporating metal oxide. They proposed that the metal oxide induced the early crystalline structure detected in the TPS films. However, the metal oxide caused an unstable crystalline structure due to low compatibility with the starch chains, and the plasticizer can quickly destroy it during the recrystallization process [41]. This result also showed that the migration of plasticizers toward the fillers highly affected the TPS’s retrogradation process. Moreover, it also showed that the retrogradation process highly influences the relationship of plasticizers with the filler in the TPS structure.

The agglomeration of plasticizer at the interfacial interaction between the NC in TPCS/2BT3NC and amylopectin will induce the amylopectin chain to form a trans crystallization structure on the surface of the NC as reported in many studies [33,34,42,43]. According to their studies, the existence of trans crystallization can be detected through the appearance of a peak at 2θ = 21.5°. The peak magnitude of TPCS/2BT3NC at 2θ = 22.2° was observed to be shifted away from 2θ = 22.5° to 21.7° from day 45 to 90 in Figure 4d. This shifting of diffraction peak was only observed for TPCS/2BT3NC90 and TPCS/5NC90. However, the TPCS/5NC90 only shows a slight shifting of peak compared to TPCS/2BT3NC90. The shifting of the peak is only seen in the films that contain NC in the structure, therefore this shifting of the peak is most probably related to the existence of transcrystalline structure in TPCS/2BT3NC90. The transcrystalline structure formed as an oriented crystalline layer of amylopectin on the NC with the inclusion of a plasticizer at the interfacial surface [44]. The transcrystalline structure in TPCS/2BT3NC may contribute to low melting enthalpy, as detected through the DSC analysis.

### 3.4. Recrystallization of Short-Chain Amylopectin in the TPCS, TPCS-C, and TPCS-HC Films Aged for 15, 45, and 90 Days as Detected through FTIR Analysis

**Figure 5:**
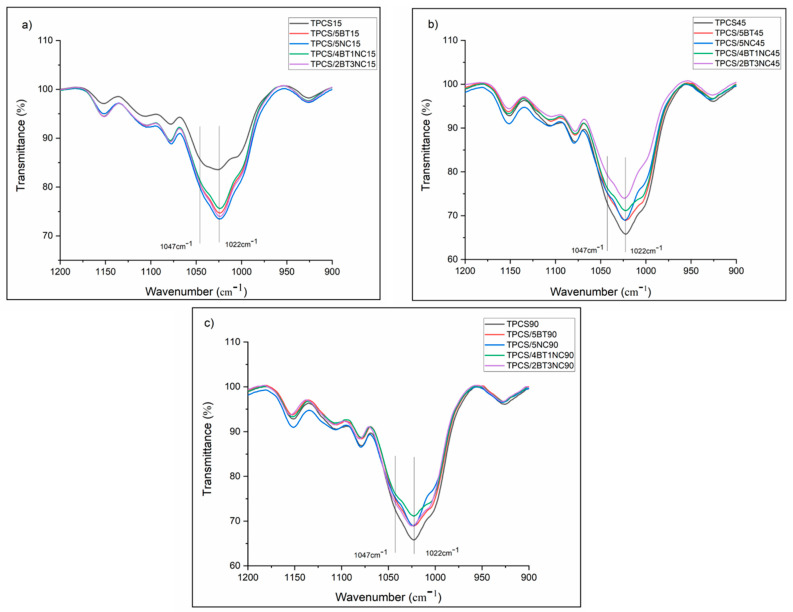
FTIR spectrum scan for neat TPCS, TPCS-C, and TPCS-HC films aged for (**a**) 15, (**b**) 45, and (**c**) 90 days from the range 900-1200 cm^−1^.

The FTIR spectra of TPCS, TPCS-C, and TPCS-HC in the range of 900–1200 cm^−1^ are shown in Figure 5. Many studies proved that the alteration of short-range molecular order could be obtained from the bands at 1022 cm^−1^ and 1047 cm^−1^ [45,46,47]. The number of amorphous regions and crystalline regions was represented by the bands at 1022 cm^−1^ and 1047 cm^−1^, respectively. The intensity ratio between the 1047 cm^−1^:1022 cm^−1^ (C values) can be used to index the starch crystalline structure region in the amorphous area. The higher the C values, the more organized the TPCS matrix structure. The infrared spectra from 900 to 1200 cm^−1^ were deconvoluted, and the 1047 cm^−1^:1022 cm^−1^ values were calculated and presented in Table 4. 

After reaching 90 days of storage, the virgin TPCS film exhibits the highest increase in the C values compared to other films, indicating that the recrystallization of the short-chain amylopectin occurred more significantly. However, by adding a single filler or hybrid fillers, the recrystallization of short-chain amylopectin could be slowed down. The C values of the TPCS/4BT1NC, TPCS/5BT, and TPCS/5NC films have been consistently increased with the increase of storage time, which is in line with the increase in crystallinity percentage measured through the XRD analysis. However, the increase of the C value for TPCS/2BT3NC from 15 to 90 days showed little difference compared to the results obtained through the XRD analysis. The crystalline percentage calculated from XRD suggested that the TPCS/2BT3NC experienced a slow rate of crystal growth from 15 to 45 days, and the growth of crystalline structure was only detected after 45 days. However, the steady increase in the C-value as detected from the FTIR analysis indicates that the TPCS/2BT3NC possessed a consistent growth of crystalline structure from 15 to 90 days. The contradiction between the FTIR and XRD results could be due to the formation of a transcrystalline structure in the TPCS/2BT3NC sample, where the recrystallization of short-range molecular was more sensitive to be detected by FTIR. The transcrystalline structure in TPCS/2BT3NC was only detected in XRD analysis by shifting the peak at 2θ. The transcrystalline does not reflect the intensity of the XRD peak because XRD is more sensitive to detecting long-range molecular crystallized structures [48].

### 3.5. Moisture Absorption of TPCS, TPCS-C, and TPCS-HC Films Aged for 90 Days

**Figure 6:**
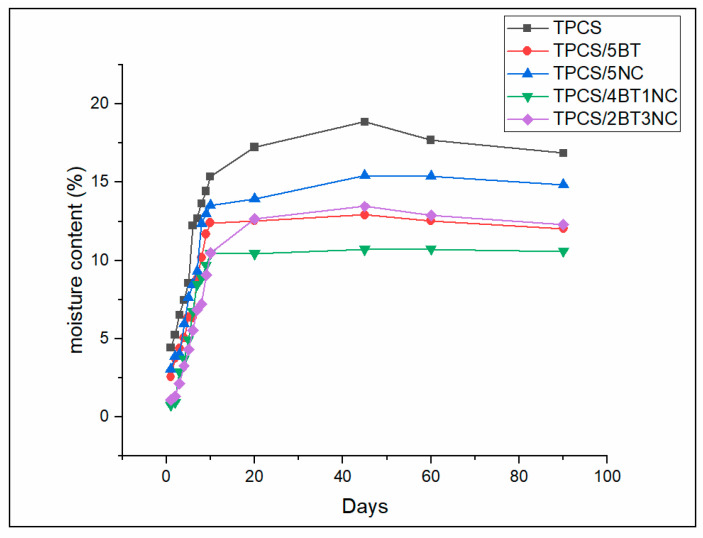
Moisture absorption percentage for virgin TPCS, TPCS-C, and TPCS-HC films aged for 90 days.

The accelerated retrogradation can happen due to the high amount of water absorption during storage and will cause the alteration of films’ crystalline structure. The changing of crystalline structure will affect the mechanical and biodegradation properties of the samples. Water can absorb into the TPCS films and accelerate the retrogradation rate. Absorbed water molecules can increase the plasticizer content in the films and may facilitate the retrogradation rate. This phenomenon has been also described by other researchers [26,27]. Figure 6 shows the moisture absorption of the TPCS, TPCS-C, and TPCS-HC films throughout the 3-month storage period. As shown in Figure 6, the moisture absorption rate of TPCS-C and TPCS-HC is lower compared to the virgin TPCS. This shows that filler could create a more tortuous path for water molecules into the films, which can reduce the TPCS films’ hydrophilic properties. All the films experienced different amounts of water absorption from the beginning of storage and reached their equilibrium moisture absorption after 10 days. The virgin TPCS film demonstrates the highest water absorption rate for the first 10 days and reached its maximum water absorption at 18.86%.

The high water absorption may be attributed to the presence of a large amount of hydroxyl groups in the starch structure, which causes a high affinity of the biopolymer to absorb water into its structure. However, after being stored for 3 months, the water absorption of the TPCS films seems to experience a slight decrease to 16.87%. This may be due to the retrogradation in the TPCS film structure, as shown in the XRD analysis, causing the expulsion of water molecules, and reducing the water content in the films. This decreasing water absorption effect was somewhat challenging to observe in the other films. TPCS/5NC has the second-highest water content compared to TPCS/5BT, TPCS/4BT1NC and TPCS/2TB3NC. This may be attributed to the higher affinity of NC toward water molecules compared to BT. However, the high crystallinity structure of nanocellulose can reduce the water sensitivity and reduce the water permeation into the TPCS films, as reported in our previous study and other studies [49,50]. Apparently, the TPCS/4BT1NC film demonstrates the lowest water absorption rate compared to other films. The low water absorption of the TPCS/4BT1NC film indicates that BT and NC, when added in the ratio of 4:1, can most efficiently repel the water inclusion as the well-dispersed particles can create a more tortuous path for the entrance of the water. 

The significantly reduced water permeability of the TPCS/4BT1NC film, when benchmarked with the virgin TPCS, could also be due to its greater aging resistance and low retrogradation rate. Fewer chain breakage, voids, and surface embrittlement in this TPCS hybrid biocomposite film make it more resistant to water absorption as it can reduce the permeation and penetration of water molecules into the films. 

## 4. Conclusions

The virgin thermoplastic corn starch (TPCS), thermoplastic corn starch composite (TPCS-C), and thermoplastic corn starch hybrid biocomposite (TPCS-HC) films were stored for 3 months to study the effect of hybrid filler on the starch retrogradation through the tensile test, DSC, XRD, FTIR, and water absorption analyses. Results indicate that the presence of single filler or hybrid fillers (nanocellulose + bentonite) can minimize the degree of starch retrogradation, thus inhibiting the aging of the TPS film. The interactions between the TPCS, plasticizer, and hybrid filler have reduced the mobility of the starch molecular chains, minimizing the rearrangement of the starch molecular chain into the crystal structure, and subsequently retarded the retrogradation process of the biopolymer. On the contrary, the absence of the hybrid filler caused the migration of the plasticizer to the film’s surface, allowing more free movement and arrangement of molecules for the formation of a more dominant amylopectin crystalline structure. This mechanism led to the retrogradation of this starch-based film.

The TPCS-C films showed moderate efficacy in reducing the starch retrogradation compared to the TPCS-HC. The TPCS-HC film is proven to possess the most negligible starch retrogradation and can maintain its mechanical stability throughout the 3-month aging period. Apparently, the ratio of nanocellulose (NC):bentonite (BT) hybrid filler in the TPCS-HC system affects the retrogradation and aging of the film during a long storing period (3 months). The high composition of the NC compared to BT in the biocomposite films can attract the migration of plasticizer molecules that accumulate in the interface of the NC and the amylopectin. This encourages the formation of the oriented crystalline layer of the amylopectin chains in the interface of the NC/amylopectin (known as a transcrystalline structure). This unstable transcrystalline region in the starch structure is responsible for the retrogradation phenomenon. 

Overall, results indicate that the TPCS film incorporating the BT:NC hybrid filler in the ratio of 4:1 is the best hybrid biocomposite sample as it has demonstrated the most stable microstructure and mechanical properties upon the 3-month storing period. This suggests that the use of a green hybrid filler of nanocellulose and bentonite with the appropriate ratio/composition can be an efficient and environmentally friendly approach to avoid early biodegradation or aging of the starch-based film in the development of a sustainable packaging material.

## Figures and Tables

**Table 1:** The formulation of TPCS, TPCS-C, and TPCS-HC films.

	Acronym	Starch (wt%)	Bentonite (BT) (wt%)	Nanocellulose (NC) (wt%)
**Virgin Thermoplastic Corn Starch (TPCS)**	TPCS	100	0	0
**Thermoplastic Corn Starch Composite (TPCS-C)**	TPCS/5BT	95	5	0
	TPCS/5NC	95	0	5
**Hybrid Thermoplastic Starch (TPCS-HC)**	TPCS/4BT1NC	95	4	1
	TPCS/2BT3NC	95	2	3

**Table 2:** Melting temperatures (Tm) and enthalpy of fusion (ΔHf) for TPCS, TPCS-C, and TPCS-HC films.

	Days					
Sample Films	15		45		90	
	T_m_ (°C)	ΔH_f_ (J/g)	T_m_ (°C)	ΔH_f_ (J/g)	T_m_ (°C)	ΔH_f_ (J/g)
TPCS	99.3	194.3	126.6	435.6	123.5	500.3
TPCS/5BT	115.0	142.6	123.5	329.6	130.3	400.5
TPCS/5NC	125.3	135.4	130.5	325.4	132.5	348.5
TPCS/4BT1NC	130.4	178.5	134.4	412.4	138.9	423.6
TPCS/2BT3NC	110.3	186.6	128.5	203.4	135.1	212.5

**Table 3:** The changes in the crystalline percentage of TPCS, TPCS-C, and TPCS-HC films after 15, 45, and 90 days of storing.

Sample	Crystallinity Percentage after Aging (%)		
	15 Days	45 Days	90 Days
TPCS	16.7	20.3	29.7
TPCS/5BT	13.5	19.4	24.4
TPCS/5NC	14.3	20.6	23.6
TPCS/4BT1NC	10.4	12.6	15.1
TPCS/2BT3NC	10.1	13.8	20.0

**Table 4:** The ratio of 1047 cm^−1^:1022 cm^−1^ (C value) of TPCS, TPCS-C, and TPCS-HC films after 15, 45, and 90 days of aging.

Days	Films	1047 cm^−1^:1022 cm^−1^ (C Value)
15 days	TPCS15	0.827
	TPCS/5BT15	0.837
	TPCS/5NC15	0.815
	TPCS/4BT1NC15	0.803
	TPCS/2BT3NC15	0.797
45 days	TPCS45	0.854
	TPCS/5BT45	0.849
	TPCS/5NC45	0.823
	TPCS/4BT1NC45	0.815
	TPCS/2BT3NC45	0.825
90 days	TPCS90	0.870
	TPCS/5BT90	0.853
	TPCS/5NC90	0.846
	TPCS/4BT1NC90	0.823
	TPCS/2BT3NC90	0.857

## Data Availability

The data presented in this study are available on request from the corresponding author.
